# Conserved spinal cord bioenergetics in experimental autoimmune encephalomyelitis in C57BL6 mice, measured using phosphorescence oxygen analyzer

**DOI:** 10.1016/j.heliyon.2021.e08111

**Published:** 2021-10-04

**Authors:** Mariam Al Shamsi, Allen Shahin, Doua Kamyan, Alanood Alnaqbi, Sami Shaban, Abdul-Kader Souid

**Affiliations:** a Department of Microbiology and Immunology, UAE University, College of Medicine and Health Sciences, Al Ain, P.O. Box 17666, Abu Dhabi, United Arab Emirates; b Department of Medical Education, UAE University, College of Medicine and Health Sciences, Al Ain, P.O. Box 17666, Abu Dhabi, United Arab Emirates; c Department of Pediatrics, UAE University, College of Medicine and Health Sciences, Al Ain, P.O. Box 17666, Abu Dhabi, United Arab Emirates

**Keywords:** Multiple sclerosis, Spinal cord, Cellular bioenergetics, Cellular respiration, Neurons, Energy conversion, Oxygen measurements

## Abstract

**Background:**

We have previously reported that spinal cord respiration (cellular mitochondrial oxygen consumption) and ATP content are conserved in the studied model of experimental autoimmune encephalomyelitis (EAE), foreseeing a recovery of the diseased rats. This exemplary lesion of multiple sclerosis is used here to measure spinal cord bioenergetics in C57BL6 mice. Our hypothesis is that, despite the well-known focal axonal mitochondrial pathology, bioenergetics of the CNS is reasonably preserved in this disease.

**Methods:**

EAE was induced with an immunodominant *m*yelin *o*ligodendrocyte *g*lycoprotein epitope in complete Freund's adjuvant, appended by injections of pertussis toxin. A low- and high-dose of the encephalitogen, administered into base of tail or hind-flank, were investigated. Control mice received only the incomplete adjuvant into tail. Oxygen measurements were based on quenching the phosphorescence of Pd(II) meso-tetra (sulfophenyl) tetrabenzoporphyrin by molecular oxygen. Cellular ATP was measured using the luciferin/luciferase system.

**Results:**

The kinetics of spinal cord oxygen consumption was zero-order (linear with time) and inhibited by cyanide, confirming oxygen was reduced by cytochrome oxidase. The rate of respiration (in μM O_2_.min^−1^.mg^−1^; measured on Days 13–28) in control mice was (mean ± SD) 0.086 ± 0.024 (n = 8) and in immunized mice was 0.079 ± 0.020 (n = 15, *P* = 0.265, Mann-Whitney test). Consistently, cellular ATP (in μmol mg^−1^ dry pellet weight; measured on Days 13–28) in control mice was 0.068 ± 0.079 (n = 11) and in immunized mice was 0.063 ± 0.061 (n = 24, *P* = 0.887, Mann-Whitney U test).

**Conclusions:**

*In vitro* measurements of spinal cord bioenergetics show conservation of the mitochondrial function in mice with EAE. These results suggest the previously documented reduced mitochondrial electrochemical potential in this disease is alterable, and likely reflects the adverse events of neuroinflammation.

## 1 Introduction

The myelin oligodendrocyte glycoprotein-induced spinal experimental autoimmune encephalomyelitis (MOG-EAE) is a frequently used rodent model of multiple sclerosis [1]. This disease affects susceptible hosts, causing infiltration of the brain and spinal cord with myelin-specific autoreactive T cells. Consequently, focal axonal inflammation, mitochondrial pathology (swollen and reduced membrane electrochemical potential), demyelination, and neurodegeneration become evident [2, 3]. These lesions are usually spontaneously reversible [2, 3].

A commonly used encephalitogen is the MOG35-55 (MEVGWYRSPFSRVVHLYRNGK) peptide in a complete Freund's adjuvant (an emulsion of water and mineral oil) that contains a strain of *Mycobacterium tuberculosis*. The immunodominant epitope and its adjuvant have been shown to activate MOG-specific Th1 cells in the periphery [4]. Infiltration of Th1 cells into the CNS is then facilitated by the pertussis toxin [5].

Several studies have shown that interactions of Th1 cells with antigen-presenting cells, such as microglia and macrophages initiate inflammatory cascades that lead to axonal damages (focal swelling and fragmentation), axonal mitochondrial derangements (including impaired NADH:ubiquinone oxidoreductase), and axonal demyelination [1, 2, 6, 7, 8]. The demyelinated and degenerative lesions have been suggested to result from an overproduction of reactive oxygen species (ROS), made mainly by oxidative bursts in activated microglia and macrophages [9].

As previously shown in a mouse model of multiple sclerosis, the damages are focal and preceded by a notable axonal mitochondrial pathology [2, 3]. The mitochondrial injury has been suggested to result from macrophage-derived ROS and nitric oxide production [2]. Consistently, neuroprotective (cytoprotective) agents can halt the progression of axon damages [2]. A reduced axonal ATP has been suggested to trigger cellular responses that lead to degenerating (necrotic) axons [10, 11]. It is unknown, however, whether the observed mitochondrial structural and functional changes are associated with permanently reduced rates of cellular respiration and ATP synthesis (a lastingly loss of axonal respiration and bioenergetics).

Cellular respiration (cellular mitochondrial oxygen consumption) refers to the mitochondrial reactions that result in oxidation of the reducing equivalents NADH/FADH_2_, with the passage of electrons to oxygen (a reduction reaction that forms ‘water of oxidation’), and the coupled ATP synthesis (phosphorylation). Impaired oxidative phosphorylation implies a blockade in these processes, including induction of apoptosis.

Reported biotechnologies for measuring dissolved oxygen include: (**1**) Electrochemical sensors (Clark electrodes, polarographic or Galvanic) that generate electrical signals in response to oxygen reduction by the polarized electrode [12]; (**2**) Optical sensors based on oxygen quenching fluorescence [13, 14, 15], phosphorescence [16, 17, 18], or luminescence; and (**3**) Micro-electrochemical techniques (scanning electrochemical microscopy) based on measurements of flow of small molecules, such as oxygen in tissues [19]. The use of ‘fluorophore-based biosensors, including solid state cartridges’ [20, 21, 22] and ‘high-resolution FluoRespirometry’ [14] have become relatively popular.

A number of recent neuroscience studies have incorporated cellular respiration as a biomarker for monitoring adverse events [23, 24, 25, 26, 27, 28] or therapeutic interventions, such as effects of cytoprotective therapies [29]. We have developed and used the previously reported methodology for oxygen measurements via phosphorescence [16] to study cellular bioenergetics in cell cultures and various tissues [17, 18]. The main principle of this analytical tool is that oxygen quenches the phosphorescence of pallidum phosphors, such as Pd(II) meso-tetra (sulfophenyl) tetrabenzoporphyrin [16]. This analytical tool (phosphorescence oxygen analyzer) has been especially useful in monitoring oxygen reactions of rapid kinetics [30] as well as oxygen measurements over several hours [31]. The applied instrument and its software development have been previously described in details [18, 32].

We have previously reported that spinal cord respiration and ATP content are well-preserved in the studied relapsing-remitting (mono-episodic with subsequent recovery) form of EAE, foreseeing the recovery of affected rats [33]. The same method is used here to measure spinal cord bioenergetics (cellular respiration and ATP content) in mice with EAE. The purpose of this work was to investigate whether *in vitro* measurements of spinal cord respiration (tissue suspended in physiologic solution) is preserved in severely diseased animals.

## 2 Materials and methods

### 2.1 Chemicals and reagents

Pd(II) meso-tetra (sulfophenyl) tetrabenzoporphyrin sodium salt (C_60_H_32_N_4_Na_4_O_12_PdS_4_, catalogue number T41161) was purchased from Frontier Scientific (Logan, UT, USA); it was dissolved in dH_2_O (2.5 mg/mL, 2 mM) and stored at -20 °C. MOG35-55 (catalogue # AS-60130-5) was purchased from AnaSpec (California, USA) as a lyophilized power (5 mg); it was dissolved in 1.0 mL dH_2_O and stored at -20 °C. *Mycobacterium tuberculosis* (MTB) strain H37Ra (catalogue # 231141) was purchased from Bio-Rad Laboratories (California, USA) as a lyophilized powder and stored at 4 °C. Freund's incomplete adjuvant (FIA, catalogue # 014H-8800) was purchased from Sigma-Aldrich (St. Louis, MO) and stored at 4 °C. Pertussis toxin was purchased from Sigma-Aldrich (St. Louis, MO) as a 50 μg lyophilized powder (catalogue #P7208), and was dissolved in 1.0 mL dH_2_O plus 2 mg/mL bovine serum albumin and stored at 4 °C. Its working solution was 6.0 μg pertussis toxin in 10 mL phosphate-buffered saline (PBS). Glucose oxidase (GO; catalogue #G7141) was purchased from Sigma-Aldrich (St. Louis, MO); its solution (10 mg/mL) was dissolved in dH_2_O and stored at -20 °C. Potassium cyanide (KCN, catalogue # 10201) was purchased from BDH Middle East LLC (Dubai, UAE); its solution was prepared in dH_2_O immediately prior to use. The remaining reagents were purchased from Sigma-Aldrich and prepared and stored as previously described [17, 18, 30, 31, 32, 33].

### 2.2 Animals

C57BL6 female mice (13 weeks old on Day 0; mean ± SD weight 20.6 ± 1.4 g, median 21.0 g, range 16.3–22.2 g) were housed at 22 °C with 60% humidity and 12-h light-dark cycles. Rodent chow and filtered water were provided *ad libitum*. It is well to know that wet rodent chow and filtered water were made always accessible to mice with all disease scores. The study was approved from the UAE University Animal Research Ethics Committee - (Ref. ERA-2019-6026; Bioenergetics of the spinal cord in mice model of chronic and relapsing-remitting EAE [experimental autoimmune encephalomyelitis]).

Sodium pentobarbital (90 μg/g, given intraperitoneally) was used for anesthesia [34]. Mice were monitored daily for weight and clinical signs of disease. Disease severity was scored as: 0, no symptoms; 1, flaccid tail; 2, hind-limb mild weakness (quick righting reflex); 3, hind-limb severe weakness (slow righting reflex); 4, hind-limb paralysis; and 5, hind-limb paralysis and partial forelimb weakness (Supplementary Videos) [2].

### 2.3 Disease induction

The following treatment protocols were employed on Day 0 (≥5 mice per group; total mice 35):

*Group 1 (adjuvant-only control mice):* Mice were injected with the adjuvant (FIA) subcutaneously into the base of tail.

*Groups 2 and 4 (lower-dose of the encephalitogen mice):* Mice were injected with 50 μg MOG35-55 plus 0.1 mg MTB subcutaneously into the base of tail or the hind-flank, respectively.

*Groups 3 and 5 (higher-dose of the encephalitogen mice):* Mice were injected with 300 μg MOG35-55 plus 0.4 mg MTB subcutaneously into the base of tail or the hind-flank, respectively.

Groups 2–5 also received 0.3 μg pertussis toxin intraperitoneally on Day 0 and 48 h later.

For Groups 2 and 4, 3.0 mL FIA was emulsified with 3.0 mg MTB and 200 μg MOG35-55; each mouse received a total of 200 μL, 100 μL on each side. For Groups 3 and 5, 3.0 mL FIA was emulsified with 12 mg MTB and 1.2 mg MOG35-55; each mouse received a total of 200 μL, 100 μL on each side.

### 2.4 Spinal tissue collection

Fragments from the spinal cord were collected as described below and shown in Supplementary Video 2. Briefly, mice were placed on a clean clip board and stabilized by pinning the four flanks. The skin of the back was cleaned with 70% ethanol, and removed to expose the bones. A cut (about 0.5 cm) was made in the lower region to expose the tip of the cord. The bones were dissected on both sides to fully expose the spinal cord. A tweezer was used to remove the entire spinal cord, which was immersed in ice-cold phosphate-buffered saline (PBS: 137 mM NaCl, 2.7 mM KCl, 4.3 mM Na_2_HPO_4_, and 1.4 mM KH_2_PO_4_, pH 7.4) supplemented with 5 mM glucose to rinse the blood. A specimen, (10–40 mg) was then *immediately* placed in an oxygen measuring vial that contained freshly prepared 2.0 mL PBS, 3 μM Pd phosphor, and 0.5% fat-free albumin with or without 5 mM glucose. The vial was sealed from air and then placed in the oxygen measuring chamber for determining the rate of respiration at 25 °C. Mixing was carried out with the aid of parylene-coated stirring bars.

The following is the supplementary data related to this article:

Supplementary Video 2 Supplementary Video 2

### 2.5 Oxygen measurement

The previously described phosphorescence oxygen analyzer was used to measure oxygen consumption by the studied spinal cord fragments [17, 18, 30, 31, 32, 33]. The Pd phosphor (absorption maximum at 625 nm; phosphorescence emission maximum at 800 nm) was used for oxygen detection. The measuring glass vials were exposed to 10 per sec light flashes from a pulsed light-emitting diode array with the peak output at 625 nm (OTL630A-5-10-66-E, Opto Technology, Inc., Wheeling, IL). Phosphorescence was detected by Hamamatsu photomultiplier tube type 932 (vacuum phototube) after passing through a filter centered at 800 nm. The amplified phosphorescence decay was digitized at 1.0 MHz by a 20-MHz A/D converter (Computer Boards, Inc., Mansfield, MA). Respiration was measured at 25 °C in 2-mL sealed glass vials.

The previously described program from Microsoft Visual Basic 6, Microsoft Access Database 2007, and Universal Library components (Universal Library for Measurements Computing Devices; http://www.mccdaq.com/daq-software/universal-library.aspx) was used for the analysis [32]. The program allowed direct reading from the PCI-DAS 4020/12 I/O Board (PCI-DAS 4020/12 I/O Board; http://www.mccdaq.com/pci-data-acquisition/PCI-DAS4020-12.aspx). Pulse detection was accomplished by searching for 10 phosphorescence intensities >1.0 volt. Peak detection was accomplished by searching for the highest 10 data points of a pulse and choosing the data point closest to the pulse decay curve [32]. The phosphorescence decay rate (1/τ) was a single exponential; I = Ae^-*t*/τ^; I = Pd phosphor phosphorescence [16]. The values of 1/τ were linear with dissolved oxygen: 1/τ = 1/τ^o^ + *k*_*q*_[O_2_]; 1/τ = the phosphorescence decay rate in the presence of O_2_, 1/τ^o^ = the phosphorescence decay rate in the absence of O_2_, and *k*_q_ = the second-order O_2_ quenching rate constant in s^−1^ μM^−1^ [16].

In the reaction vial (sealed from air), oxygen concentration decreased linearly with time, indicating the kinetics of cellular mitochondrial oxygen consumption was zero-order [17]. The rate of respiration (*k*, in μM O_2_ min^−1^) was thus the negative of the slope of d[O_2_]/d*t*. The calibration reactions contained PBS with 3 μM Pd phosphor, 0.5% fat-free albumin, 50 μg/mL glucose oxidase (catalyzed the reaction: D-glucose + O_2_ → D-glucono-δ-lactone + H_2_O_2_) and various concentrations of β-glucose [16, 17, 18]. Potassium cyanide (KCN, approximately 10 mM, prepared in dH_2_O) inhibited respiration, confirming oxygen was reduced in the mitochondrial respiratory chain [33].

### 2.6 ATP determination

ATP concentration was measured using the Enliten ATP Assay System (Bioluminescence Detection Kit, Promega, Madison, WI) as previously described [33]. Briefly, extracted spinal fragments were immediately vortexed vigorously and homogenized in 0.5 mL ice-cold (freshly prepared) 2% trichloroacetic acid for 3 min; the supernatants were then collected by centrifugation (1000x*g* at 4 °C for 10 min) and stored at -80 °C. For the analysis, a 0.1 mL aliquot of the sample was neutralized with 0.9 mL of 100 mM Tris-acetate and 2 mM EDTA, *p*H 7.75 (final *p*H, about 8). The luminescence intensity was measured at 25 °C using Glomax Luminometer (Promega, Madison, WI) and following the manufacturer's instructions [33]. The ATP standard curve was linear from 0.05 pmol to 0.5 pmol (*R*^*2*^ = 0.9599). The results are expressed as μmol ATP per mg dry pellet weight.

### 2.7 Statistical analysis

Data were analyzed using SPSS statistical package (version 20). The nonparametric test (2 independent variables, Mann-Whitney U test, asymptotic 2-sided significance) was used to compare between two groups. The nonparametric test (Kruskal Wallis test, asymptote significance) was used to compare between three or four groups. Two-tailed tests were used. *P* ≤ 0.05 was considered significant.

## 3 Results

### 3.1 Spinal cord cellular respiration

**Figure 1:**
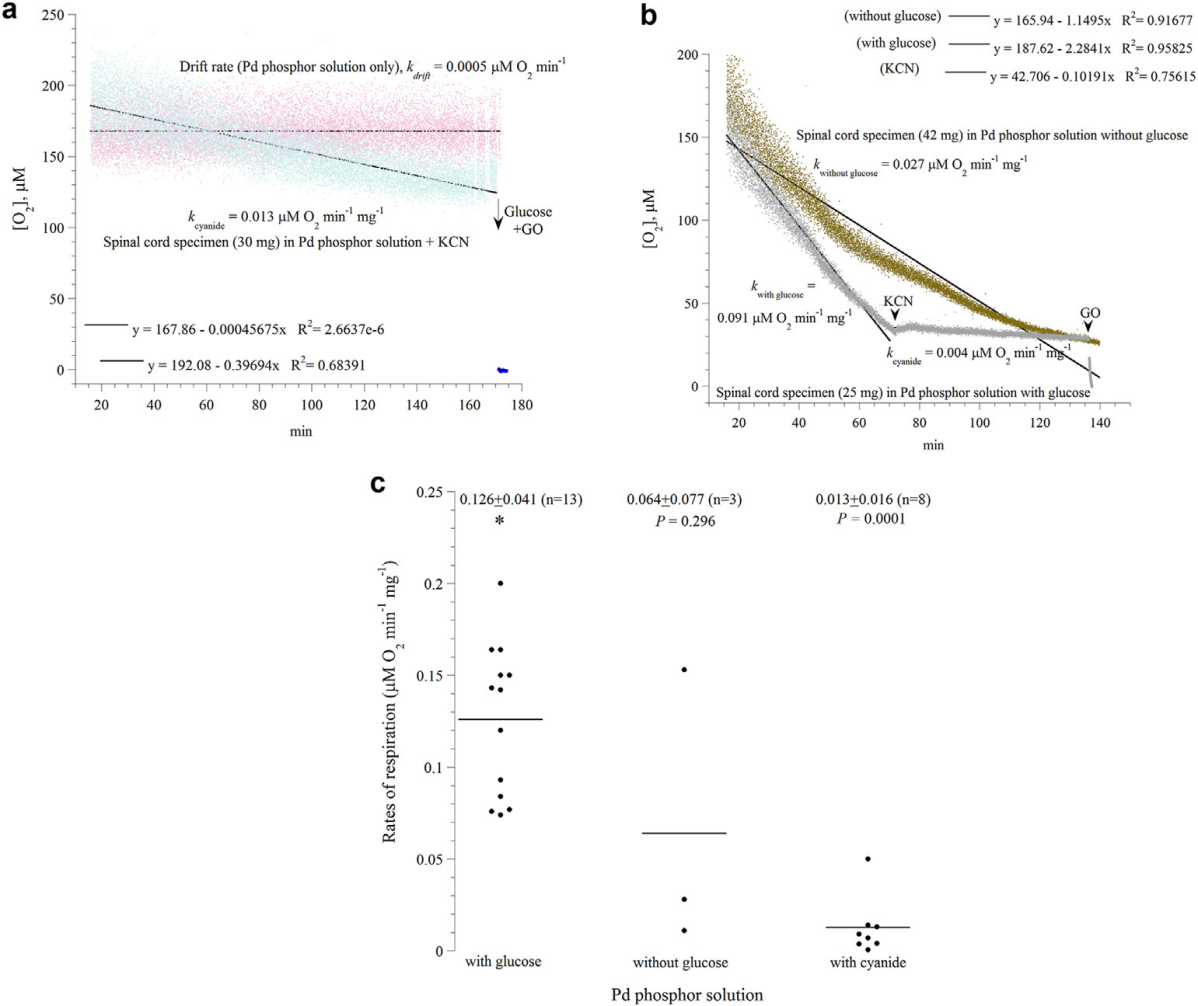
*Representative runs of spinal cord cellular respiration*: *Panel a*: Drift rate (*k*_drift_) in Pd phosphor solution only (without added tissue). Also shown is spinal cord respiration (*k*_cyanide_) of a 30-mg specimen in Pd phosphor solution plus KCN (cyanide insensitive respiration). *Panel b*: Spinal cord respiration in Pd phosphor solution with 5 mM glucose (a 25-mg specimen; respiration driven by endogenous nutrients plus added glucose) or without added glucose (a 42-mg specimen; respiration driven by endogenous nutrients only). The additions of potassium cyanide (KCN) and glucose oxidase (GO) are also shown (arrow heads). KCN halted the decline in oxygen concentration with time, confirming oxygen was reduced by cytochrome oxidase. In these experiments, the mice were sacrificed at minute zero. The lines are linear fits; the linear equations are shown. The rate of respiration (in μM O_2_ min^−1^) were set as the negative of the slopes of linear lines. The values of *k* (expressed in μM O_2_ min^−1^ mg^−1^) are normalized for the sample weight differences. As shown, the noise level over the duration of the experiments (about 3 h) is negligible. *Panel c*: Summary of the measured rates of respiration in Pd phosphor solution with glucose, without glucose, or with cyanide. Mean ± SD (n) of the rates of respiration are shown. The values of *P* are Mann-Whitney U test.

Representative runs of four different experimental conditions are shown in Figure 1a-b. The *first* run is in ‘Pd phosphor solution only’ (without added tissue), which demonstrates a negligible drift rate over three hours (Figure 1a). Thus, correction for the noise level was unnecessary. This length of phosphorescence signal stability allows an extended monitoring, such as measuring cellular respiration in small samples over several hours. The *second* run represents spinal cord respiration of a 30-mg specimen in ‘Pd phosphor solution plus potassium cyanide’ (KCN, a specific inhibitor of cytochrome oxidase and a well-known neurotoxin), Figure 1a. The rate in the presence of cyanide is relatively small (*k*_cyanide_ = 0.013 μM O_2_ min^−1^ mg^−1^), reflecting the ‘cyanide insensitive respiration’ (Figure 1a). Inhibition of respiration by cyanide confirms oxygen is consumed (reduced) by cytochrome oxidase (Complex IV of mitochondrial respiratory chain). The addition of ‘β-glucose plus glucose oxidase’ demonstrates the halt of oxygen consumption occurred in the presence of oxygen (Figure 1a). It is worth noting that the signal in the presence of ‘β-glucose plus glucose oxidase’ corresponds to the true ‘zero dissolved oxygen concentration’ (Figure 1a). The *third* run is spinal cord respiration of a 42-mg specimen in ‘Pd phosphor solution without added glucose’ (i.e., respiration driven by endogenous cellular nutrients, Figure 1b). Its profile fits exponential curve (*R*^*2*^ = 0.99162) better than linear curve (*R*^*2*^ = 0.89793), likely due to limited nutrients especially after about 50 minutes. The *fourth* run was spinal cord respiration of a 25-mg specimen in ‘Pd phosphor solution plus 5 mM glucose’ (i.e., respiration driven by endogenous cellular nutrients plus cellular influx of the added glucose, Figure 1b). This profile is linear (*R*^*2*^ = 0.94969), and its rate is about 3-fold faster than that without glucose (0.091 versus 0.027 μM O_2_ min^−1^ mg^−1^, Figure 1b). Thus, the presence of glucose has improved spinal cord respiration (tissue viability) quantitatively (faster rate) and qualitatively (improved linear kinetics). It is well to know that the linear decline in oxygen concentration with time reflects the expected zero-order kinetics of oxygen reduction by cytochrome oxidase (a constant amount of oxygen is consumed per minute) and signifies viable samples. Again, the addition of cyanide halted cellular respiration, and the addition of glucose oxidase depleted the remaining oxygen in the solution (Figure 1b). Summary of all results (respiratory rates, expressed in μM O_2_ min^−1^ mg^−1^) is shown in Figure 1c. The rate of respiration without cyanide was 0.126 ± 0.041 (n = 13) and with cyanide was 0.013 ± 0.016 (n = 13); about 90% inhibition by cyanide (*P* = 0.0001).

### 3.2 Repeated runs of the same spinal cord specimen over six hours

**Figure 2:**
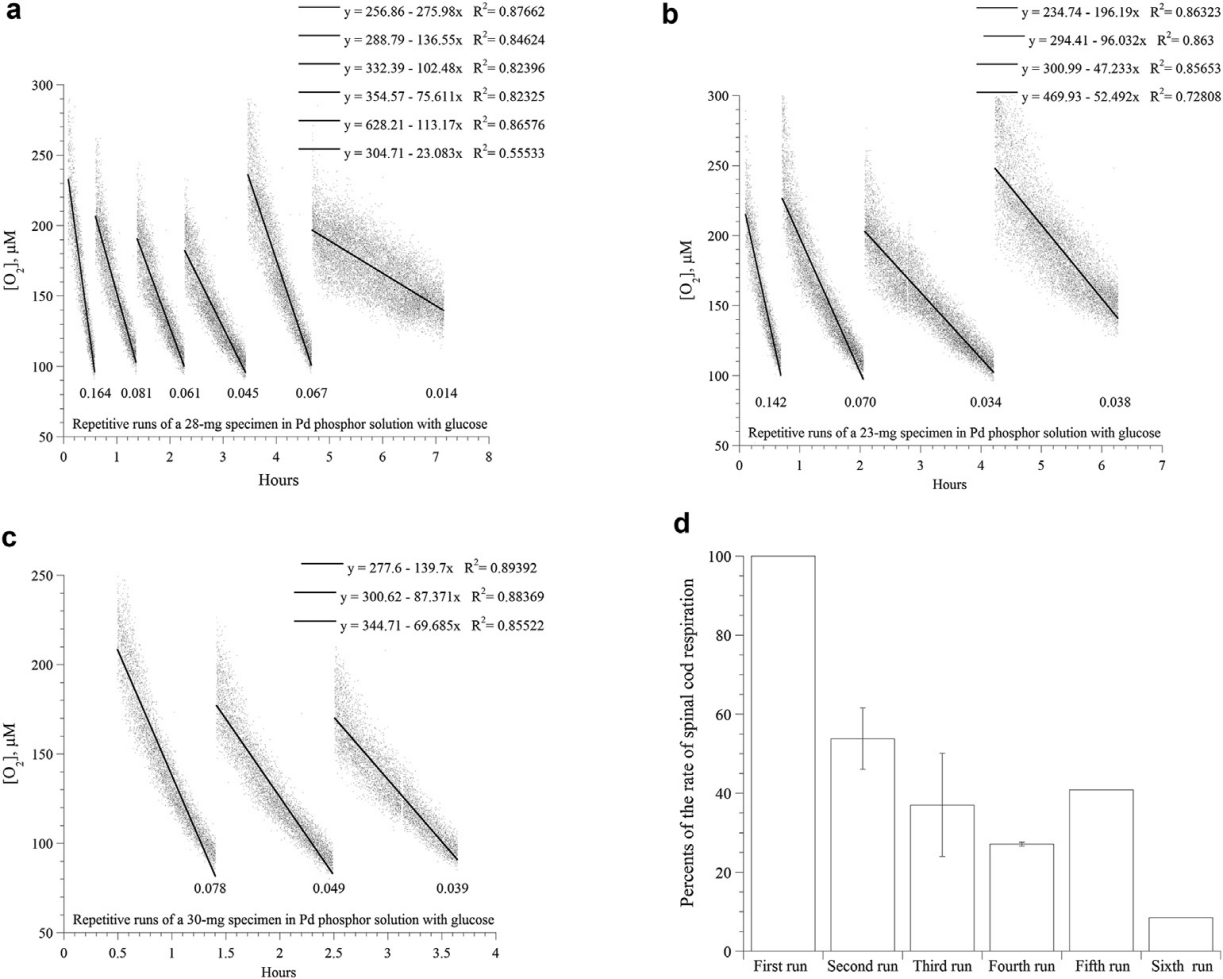
*Repeated runs of spinal cord specimen over six hours.* Three independent experiments are shown. *Panels a-c*: For each experiment, at an oxygen concentration of about 100 μM, the specimen was transferred to a new vial containing a freshly prepared, air-saturated Pd phosphor solution with 5 mM glucose. This process was repeated a few times as shown. The lines are linear fits; the linear equations are also shown. The rate of respiration (in μM O_2_ min^−1^ mg^−1^; normalized for the sample weight difference) is shown at the bottom of each run. *Panel d*: Summary of the decline in the rate of spinal cord respiration with the repeated runs. Error bars are standard deviation.

Three independent experiments are shown (Figure 2a-c). In these experiments, the reaction mixture was reaerated by transferring the spinal cord specimen to a freshly-prepared ‘Pd phosphor solution plus 5 mM glucose’ when oxygen concentration reached about 100 μM. This procedure was repeated a few times as shown. The overall results of these experiments are summarized in Figure 2d. As shown, reasonable tissue viability was maintained over the duration of these experiments.

### 3.3 Weight changes are functions of the encephalitogen dose and route of administration

**Figure 3:**
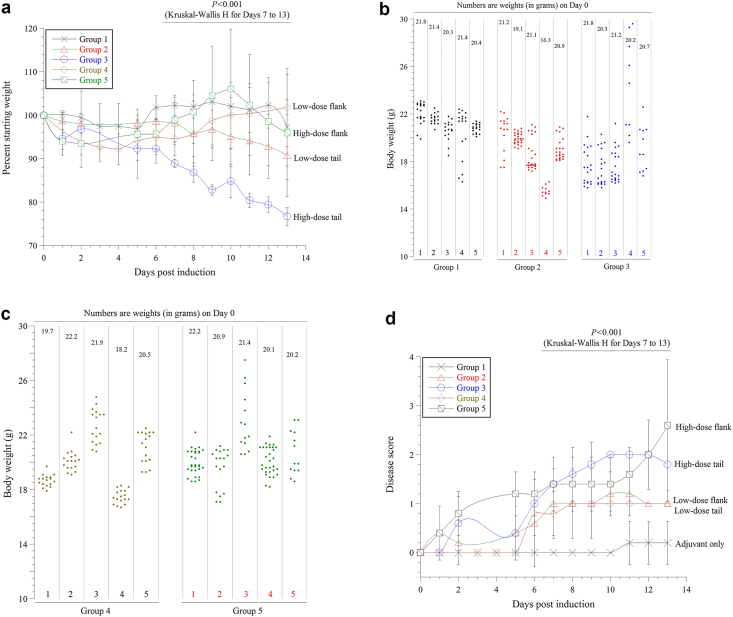
Weight changes (Panels a–c) and disease scores (Panel d) in the studied mice (≥5 mice per group, total 33 mice). *Panel a*: Linear plot of weight changes from Day 0 to Day 13. Values (mean ± SD) are percentages of the daily weight divided by the starting weight. *P* (Kruskal Wallis test) is for the average weights between Groups 2 to 5 from Day 7 to Day 13. *Panels b-c*: Dot plots of the daily weight of each mouse from Day 0 to Day 28 (‘censored data’). Each column shows the daily weights of an individual mouse (each dot is the weight on a particular day). Wide dispersions signify large weight changes (e.g., Group 2/mouse 1), while narrow dispersions signify small weight changes (e.g., Group 2/mouse 4). *Panel d*: Linear plot of disease scores from Day 0 to Day 13. Values are mean ± SD of the daily disease scores. *P* (Kruskal Wallis test) is for the average scores between Groups 2 to 5 from Day 7 and Day 13.

In order to comprehend the clinical impacts of EAE, weight was monitored over 28 days. Figure 3a is a linear plot of the weight changes between Days 0 and 13, and Figure 3b-c are dot plots of the ‘censored weight changes’ for Days 0–28.

Various degrees of initial weight loss (within Days 1 and 5) were present in all groups, including the mice who received incomplete adjuvant only (Group 1). Subsequently, a marked weight loss was evident in the mice who received the encephalitogen in the base of tail (Groups 2–3), especially with the higher dose (Group 3, averaging 23% on Day 13), *P* < 0.001. The weight loss in Group 4 (low-dose encephalitogen in hind-flank) was relatively brief and recovered by Day 10. In contrast, the mice who received high-dose encephalitogen in hind-flank (Group 5) showed a weight gain that peaked on Day 10.

Group 3/mouse 4 developed an excessive weight gain of 47% due to generalized edema (Figure 3b) and died on Day 10. Group 5/mouse 3 also developed edema (29% weight gain) on Day 10 (Figure 3c); its weight, however, returned to the baseline on Day 14. Thus, edema appears to be an adverse event of the higher immunogen dosing, and future studies are required to understand its pathogenesis.

### 3.4 Clinical severities are also functions of the encephalitogen dose and route of administration

We next compared the clinical severity of EAE among treated mice (Figure 3d). The onset and severity of the disease also varied significantly between Groups 2 to 5 (*P* = 0.001, Kruskal Wallis test). Overall, the disease score progressed from Days 7–13, and was more prominent in mice who received the higher dose of the encephalitogen compared to those who received the lower dose (*P* = 0.001, Mann-Whitney U test) regardless of the administration route. The onset of disease was also earlier in the mice who received the higher dose.

It is well to note that three mice in Group 5 and one mouse in Group 2 reached a disease score of 4 between Days 14 and 17. None of the studied mice, however, showed signs of disease improvement or recovery within the 28 days. One mouse in Group 1 (received the adjuvant only) developed a weak tail (disease score 1) from Days 11–21.

### 3.5 Preserved spinal cord bioenergetics

**Figure 4:**
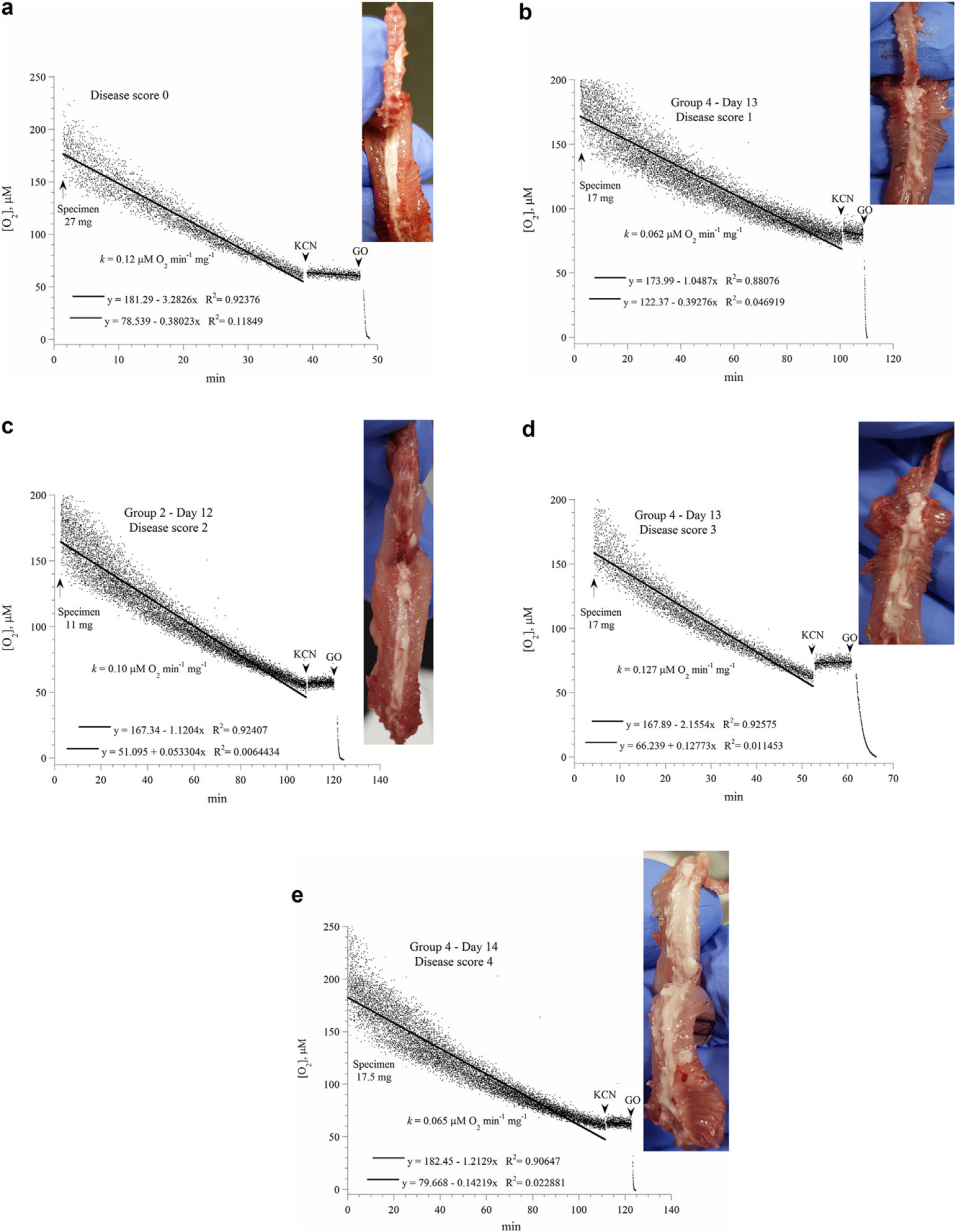
*Spinal cord respiration*. Representative runs of spinal cord respiration of mice with noted disease scores (Panels a-e). Spinal specimens were placed in PBS plus 5.0 mM glucose. The lines are linear fits. The additions of potassium cyanide (KCN) and glucose oxidase (GO) are shown. The values of *k* (in μM O_2_ min^−1^ mg^−1^) were set as the negative of the slopes of the shown upper linear equations. The lower linear equations were in the presence of KCN. The noise level over three hours was negligible (not shown). Spinal cords of these studied mice are shown as inserts. Mice were sacrificed at minute zero. The spinal cords of the treated mice were disjointed and adhered to the bones (difficult to dissect in one piece).

**Figure 5:**
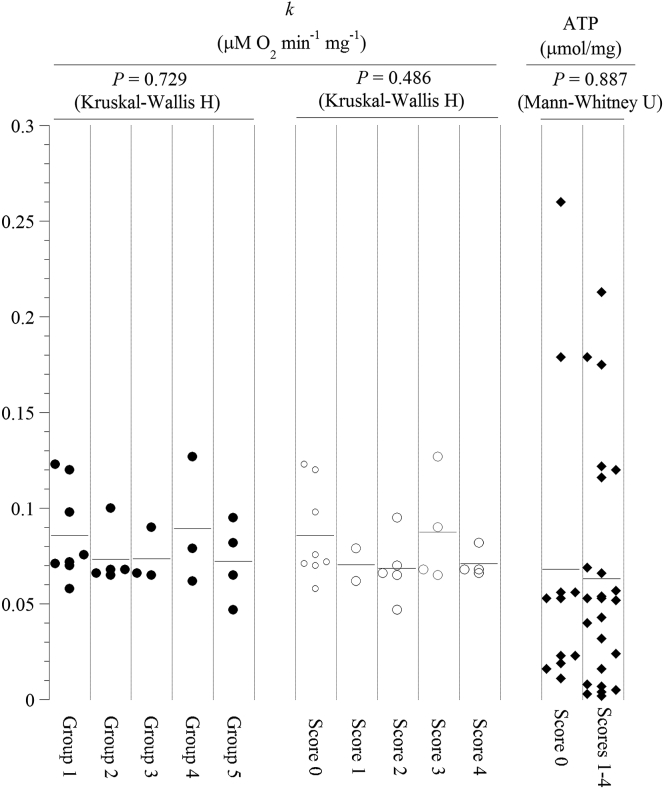
Dot plot summarizing the results of spinal cord respiration (values of *k*) sorted by experimental groups and disease scores. Horizontal lines are mean.

To test our hypothesis that spinal cord bioenergetics is preserved in EAE, cellular respiration and ATP content were measured in spinal cord specimens from mice with different disease scores. Representative runs of cellular respiration are shown in Figure 4a-e, and a summary of the results is shown in Figure 5. The decline in oxygen concentration with time was linear, reflecting the expected zero-order kinetics of oxygen reduction by cytochrome oxidase (a constant amount of oxygen being consumed per min). The addition of cyanide halted about 90% of oxygen consumption, confirming oxygen was reduced by cytochrome oxidase. The addition of glucose oxidase depleted the remaining oxygen in the solution. The rate of spinal cord respiration (*k*, in μM O_2_ min^−1^ mg^−1^, measured on Days 13–28) was not significantly different among the studied groups (Figure 5). Overall, the value of *k* (mean ± SD) in control mice was 0.086 ± 0.024 (n = 8) and in symptomatic immunized mice (disease score of ≥1) was 0.079 ± 0.020 (n = 15, *P* = 0.265, Mann-Whitney test). Cellular ATP (μmol mg^−1^ dry pellet weight; measured on Days 13–28) in control mice was 0.068 ± 0.079 (n = 11) and in immunized mice was 0.063 ± 0.061 (n = 24, P = 0.887, Mann-Whitney U test), Figure 5.

### 3.6 Spinal cords in the studies mice with EAE

Importantly, spinal cords of mice with disease scores of 1–4 were visually abnormal, showing swellings, loss of integrity, tight adherence to bones, and occasional fragmentations (Figure 4, inserts). These visible changes are consistent with the known focal inflammatory changes in this rodent model of multiple sclerosis [2, 3].

## 4 Discussion

The above described approach allows investigating the effects of cytoprotective (e.g., *N*-acetylcysteine and other sulfhydryl-containing molecules) and neuroactive compounds on spinal cord viability *in vitro*. It would also permit studying the adverse effects of neurotoxins (e.g., rotenone) and CNS diseases (including inflammatory and neurodegenerative disorders). Advantages of this methodology include the stability of Pd phosphors and their rapid phosphoresce signals (allowing monitoring fast reactions, such as molecules carried by nanoparticles). Tissues with high metabolic requirements, such as neurons are especially vulnerable to mitochondrial damages [35]. Consistently, mitochondrial dysfunction has been associated with several CNS pathologies, including the neurodegenerative diseases [36]. For these disorders, cellular mitochondrial oxygen consumption has been adopted as a biomarker for assessing the cellular bioenergetics. This sensitive analyte is altered in many CNS diseases, including Alzheimer (Complex IV), Parkinson (Complex I) and Huntington (Complexes II and III) [37, 38, 39].

A number of methods have been used for measuring cellular mitochondrial oxygen consumption in neuroscience (reviewed in references [40, 41]). Typically, these analytical tools are applicable to cell cultures, cell suspensions, tissue fragments, tissue homogenates in suspension, and isolated mitochondria. Briefly, the multi-well plate reader Seahorse XFe24 Analyzer (Agilent Technologies) and Oroboros O_2_k-FlouroRespirometer (Oroboros Instruments) are, to some degree, more advanced than the conventional, low-cost Clark oxygen electrode chambers. Nevertheless, these instruments have similar biologic applications and provide comparable yields. The ‘phosphorescence oxygen analyzer’, on the other hand, has significant advantages over these methodologies. These benefits include simplicity of the measurements, stability of the Pd phosphors in solutions, low-cost (an estimated cost of multiple daily experiments is about $100 per year), reliability and reproducibility of the signals, applicability to studying rapid oxygen reactions (allowing ten oxygen concentration measurements per second), and suitability for monitoring processes over many hours or even days. Future studies are needed to compare the phosphorescence oxygen analyzer with other available techniques.

The main research question in this study is whether *in vitro* measurements of spinal cord respiration (spinal cord specimens suspended in physiologic solution) is preserved. The results here confirm an unaltered *in vitro* spinal cord respiration in mice with severe EAE. Thus, the observed mitochondrial dysfunction (mainly reduced inner membrane electrochemical potential) in the previous studies likely reflect adverse events of the neuroinflammation (e.g., inhibition of cellular bioenergetics by intra- and extracellular disease-specific mediators), rather than an irreversible mitochondrial damages [2, 3]. This view is consistent with observations from a single patient, suggesting “inflammation alone may be sufficient to cause significant clinical deficits” [42].

In this study, the spinal cord bioenergetics was assessed in groups of mice who received two different encephalitogen doses through two different routes of administration. These treatments produced various clinical severities and forms of the disease. Nevertheless, the results are consistent among the studied groups and disease scores (Figure 5), showing a reasonable conservation of the spinal cord respiration and ATP content, accounting for the known reversible course of the axonal damage in this disease. The results are also consistent with our previous findings in the rat model of EAE, showing a reasonably preserved spinal cord bioenergetics in this disease [33].

A few points however require special emphasis. *First*, the *in vitro* results shown here may not correlate with the *in vivo* status of spinal cord metabolism within cerebrospinal fluid (CSF) that contains inflammatory mediators and their neurotoxic products. Therefore, future studies are needed to determine the effects of the CSF from diseased mice on *in vitro* measurements of spinal cord respiration.

*Second*, the previously reported mitochondrial pathology (swollen) and reduced inner membrane electrochemical potential were in focal axon lesions in a spontaneously reversible disease [2]. Our results here show reasonable cellular mitochondrial oxygen consumption and ATP content in the spinal cords despite these previously described disrupted mitochondria [2, 3]. Feasible explanations of the overall findings may include a compensatory increase in the function of intact axonal mitochondria, trafficking of mitochondria into focal axon lesions, and upregulation of the aerobic metabolism [43]. It is important to note that a reversible disease is expected to be coupled to sufficient mitochondrial function [44].

*Third*, a large intra- and inter-group variations in the studied model of EAE are noted here. Their sources are unknown and their resemblance to the clinical spectrum of multiple sclerosis are also unclear. The need to systematically characterize and standardize the rodent model of EAE is clear. This task allows a more effective extrapolation of the results to the human disease multiple sclerosis. Moreover, disease score does not necessary reflect the severity of the CNS lesions. For example, inspecting the spinal cords clearly distinguishes between disease score 0 and disease scores ≥1. However, the spinal cord appears the same for scores 1 to 4. Therefore, objectives measures of the clinical disease are necessary. An infrared-based automated activity monitoring system has been recently proposed for this purpose [45].

*Fourth*, 25 °C is more suitable than 37 °C for the measurement of spinal cord cellular respiration *in vitro*. As shown in Figure 1b, the relatively rapid intracellular nutrient depletion at 25 °C (about 40 min) would have been much faster at 37 °C. Figure 2 shows the respiration was reasonably preserved at 25 °C, allowing an accurate determination of its zero-order rates. This inference was evident by the linearity of oxygen consumption with time. It is also well to note that this experimental design is especially important for the measurements made on fragments of the central nervous system, in comparison to those made on cell suspensions. Thus, the union of ‘lower temperature’ and ‘smaller sample size’ is essential for the accurate determination of spinal cord respiration. Furthermore, the colorless solution PBS is more suitable than the cell culture media (usually red-colored that is known to change with time) for phosphorescence measurements that utilizes a red light (625 nm) for excitation.

It is worth noting that incorporating the ‘cyanide-sensitive respiration rate’ would have no effects on the final results. Nevertheless, the shown cyanide-insensitive rates allow such calculations if desired. *Fifth*, autopsies on the rodents died of complications of EAE may reveal valuable clinical information.

The results here do not exclude mitochondrial pathology in EAE [46]. They rather explain that the overall spinal cord bioenergetics in EAE remain reasonably intact, allowing repair of the axonal damages. Therefore, therapeutic efforts should focus on blocking the inflammatory cascades early in the disease process in order to minimize the spinal cord injury. An overwhelming axonal damage in EAE may overcome the cellular repair capacity. As emphasized previously [2], the disease is reversible and combined molecularly targeted therapies may be needed to overcome the noted pathology in this disease.

It is important to note that the reliability and reproducibility of cellular ATP determination are hindered by the rapid cellular ATP hydrolysis before the sample is quenched in the trichloroacetic acid. Therefore, collecting and processing samples for determining ATP quantity require special measures to ensure consistency and efficiency. Although these efforts were implemented here, the variation remained high. Due to this concern, it is preferable to measure the rate of cellular ATP synthesis rather than cellular ATP content. The rate cellular ATP synthesis can be than compared with that of cellular respiration (coupled oxidative phosphorylation).

## 5 Conclusions

A simple, reliable and reproducible approach for determining oxygen consumption by murine spinal cord specimens *in vitro* is described in this study. The endogenous nutrients of spinal cord can support cellular respiration for about 30 min. Thereafter, a continual respiration requires added glucose. In the presence of glucose, the kinetics of spinal cord respiration is linear with time (zero-order kinetics) and is inhibited by cyanide. Respiration can be applied as a biomarker for assessing CNS in health and disease. The described method is also applicable to investigating animal models for various nervous system diseases (as discussed in this study), and exploring effects of neuroactive compounds on spinal specimens *in vitro*. The results show preserved spinal cord respiration and ATP content in severe EAE. Interventions need to primarily focus on molecularly targeted therapies that block the intracellular inflammatory cascades in this neuroinflammatory disease of the CNS. In addition, strategies that block the extracellular inflammatory mediators are also needed.

## Declarations

### Author contribution statement

Mariam Al Shamsi: Conceived and designed the experiments; Performed the experiments; Analyzed and interpreted the data; Wrote the paper.

Allen Shahin: Performed the experiments; Analyzed and interpreted the data; Contributed reagents, materials, analysis tools or data.

Doua Kamyan, Alanood Alnaqbi: Performed the experiments; Analyzed and interpreted the data.

Abdul-Kader Souid: Conceived and designed the experiments; Analyzed and interpreted the data; Wrote the paper.

### Funding statement

This work was supported by a grant from the College of Medicine & Health Sciences, UAE University.

### Data availability statement

Data included in article/supplementary material/referenced in article.

### Declaration of interests statement

The authors declare no conflict of interest.

### Additional information

No additional information is available for this paper.

## Appendix A Supplementary data

RR-SC score 1 RR-SC score 1

Score 0 Score 0

CH-Flank score 4 CH-Flank score 4

CH-SC score 2 CH-SC score 2

CH-SC score 3 CH-SC score 3
